# 

**Published:** 1914-04-04

**Authors:** 


**Doctors' Wives Association.**

(*See page* 8.)

**COUPON.**

I hereby certify that I am a doctor's wife, and I beg to send you my name and address as one who wishes to become a member of the proposed Doctors' Wives Association. I hereby agree to qualify as a member by paying the subscription of 7s. 6d. per annum on or before May 1, 1914, such subscription entitling me each year to a free copy of The Hospital newspaper delivered weekly, with the Doctors' Wives section, and to include my annual subscription to the Association.

This agreement undertaking to subscribe on my part is subject to at least 1,000 doctors' wives conditionally joining the proposed Association by signing and returning to "Co-operation" as below by April 18 next a similar coupon to that to which I hereby attach my signature.

(Signed) ....................................

(Wife of) * ....................................

(Address) ....................................

....................................

(Date) ....................................

*Note.*—When filled in and signed cut out this coupon, place it in an envelope and address and post it to:

Co-Operation,
c/o The Scientific Press,
28 & 29 Southampton Street, Strand,
London, W.C.

* Here insert full name and qualifications.

### Appointments.

Dr. H. W. Parnis has been appointed sixth assistant medical officer at Colney Hatch Mental Hospital.

Mr. Ivor Joynt, B.C., has been appointed clinical assistant to the out-patients' physicians at the West End Hospital.

Mr. F. Vincent Denne, M.D. (Brux.), M.R.C.S., L.R.C.P., L.S.A. Lond., L.D.S. Eng., has been appointed dental surgeon to the West End Hospital for Nervous Diseases, in the room of Mr. J. Sim Wallace, M.D., D.Sc., retired.

Mr. Adair Dighton, M.B. (Edin.) and F.R.C.S. (Edin.), has been appointed clinical assistant to the out-patients' physicians at the West End Hospital. Mr. Adair Dighton holds the appointments of honorary assistant surgeon to the Eye and Ear Infirmary, Liverpool, honorary ophthalmic and aural surgeon to the Birkenhead Children's Infirmary, and honorary ophthalmic and aural surgeon to the Toxteth Infirmary.

Dr. R. G. Riches, assistant medical superintendent at Camberwell Infirmary, has resigned his appointment. The Board has resolved to advertise for applicants for the vacant position, and to communicate with the Scholastic, Clerical and Medical Association. They have also decided to grant Dr. Riches a suitable and satisfactory testimonial.

Dr. Llewellyn Warburton, M.R.C.S., L.R.C.P., has been appointed by the Camberwell Board of Guardians second assistant medical officer at the Infirmary and Gordon Road Workhouse. He was previously house surgeon at the Royal Infirmary, Sheffield, and medical officer at sea on the Deep Sea Fisheries Mission Ship *Alpha*. Dr. Lionel G. Pearson, M.B. (Durham), has been appointed third assistant medical officer. His previous appointments include those of senior house surgeon, Beckett Hospital, Barnsley, and house physician to the Leeds General Infirmary.

### Gifts and Bequests.

To commemorate the silver wedding of the Duke and Duchess of Portland and the coming of age of their eldest son, the Marquess of Titchfield, the Duke has sent a cheque for £250 to the Nottingham General Hospital and £100 to Newark Hospital.

Mrs. Margaret Jury, of Donnybrook, Dublin, has left £200 to St. Joseph's Blind Asylum, Dublin, and £50 to St. Joseph's Asylum, Dublin.

Mr. Thomas Rickman Harman, of Sindlesham House, Wokingham, Berks, has left £1,000 to the Royal Berkshire Hospital.

RATES OF SUBSCRIPTION: Twelve months, 6/6; Six months, 4/-; Three months, 2/-, post free to any address in the United Kingdom. Abroad, Twelve months, 8/8; Six months, 5/-; Three months, 2/6, post free.—Address The Subscription Dept., "THE HOSPITAL," The Hospital Building, 28/29 Southampton Street, Strand, London, W.C.

